# Ethical principles, challenges and opportunities when conducting genetic counselling for schizophrenia

**DOI:** 10.3389/fpsyt.2023.1040026

**Published:** 2023-06-21

**Authors:** Zukiswa Zingela, Funeka Sokudela, Yanga Thungana, Stephan van Wyk

**Affiliations:** ^1^Executive Dean’s Office, Nelson Mandela University, Port Elizabeth, South Africa; ^2^Department of Psychiatry, University of Pretoria, Pretoria, South Africa; ^3^Department of Psychiatry and Behavioural Sciences, Walter Sisulu University, Mthatha, South Africa

**Keywords:** schizophrenia, ethics, challenges, opportunities, genetic, counselling, under-resourced community

## Abstract

Ethical challenges of genetic counselling for schizophrenia include effective communication of critical scientific information in an easily understood manner by patients and relatives, and the ability to ensure communication is unencumbered by medical jargon. Levels of literacy in the target population may limit this process, making it difficult for patients to attain the desired levels of informed consent to make crucial decisions during genetic counselling. Multilingualism in target communities may further complicate such communication. This paper outlines the ethical principles, challenges and opportunities facing clinicians when conducting genetic counselling for schizophrenia and how these might be met, drawing on lessons from South African studies. The paper draws on reflections of clinician and researcher experiences gained from clinical practice or research on the genetics of schizophrenia and psychotic disorders in South Africa. The context of genetic studies in schizophrenia is used to illustrate the ethical challenges in genetic counselling for schizophrenia, both in clinical and research settings. Attention is also drawn to multicultural and multilingual populations, particularly where the preferred language lacks a well-developed scientific language of communication for some of the genetic concepts that have to be presented during the genetic counselling process. The authors describe the ethical challenges and how to address these to empower patients and relatives to make well-informed decisions despite these obstacles. Principles applied by clinicians and researchers during the genetic counselling are described. Potential solutions, including the establishment of community advisory boards to address potential ethical challenges inherent to the genetic counselling process, are also shared. Genetic counselling for schizophrenia still faces ethical challenges which require a balance of principles of beneficence, autonomy, informed consent, confidentiality and distributive justice, while striving to present accuracy in the science that guides the process. Evolution in language and cultural competency therefore needs to occur alongside scientific advances in genetic research. Key stakeholders need to partner and build capacity and expertise in genetic counselling through the provision of funding and resources. The goal of partnerships is to empower patients, relatives, clinicians and researchers to share scientific information in a manner guided by empathy while retaining scientific accuracy.

## Introduction

Genetic counselling in schizophrenia is an essential part of clinical practice but needs to be evidence-based and guided by developments in genomic research. Research into the genetic causes of schizophrenia, and other serious illnesses, may include relatively large sample sizes and data sharing across international borders (1–4). Ethical principles, concepts and challenges that arise during the processes that govern genetic research may overlap challenges that manifest during the genetic counselling for schizophrenia itself. Examples of studies on the genetics of psychosis, schizophrenia and other disorders include the Genetics of Schizophrenia in the South African Xhosa (SAX) Study (1), the Neuropsychiatric Genetics of African Populations-Psychosis (NeuroGap-Psychosis), and the Human Heredity and Health in Africa (H3Africa) initiative (2, 3). Data from these studies was from large sample sizes, across different populations spanning many countries. If one accepts the importance of diversity in sampling genetic data as a means of expanding our knowledge of the role of genetics in psychiatric diagnoses like schizophrenia, then such cross-country approaches to genetic research and genetic counselling are crucial for proper representation of different populations (3–6). Learnings from under-resourced populations which face chronic funding constraints, in the face of data mining and lack of reinvestment in expertise on genetics, underscore the added complexity of ethical principles that need to be considered during genetics studies (4).

A relevant question that arises when considering the process of genetic counselling is whether the ethical framework that governs this type of counselling and the sharing of information on genetic results is robust enough to ensure the ethical conduct of clinicians while protecting the rights of patients. The Human Heredity and Health in Africa Consortium (H3Africa) Ethics and Community Engagement Working Group identified some of the principles pertaining to genomics research in Africa and established guidelines for consideration of feeding back genetic results of health importance (7). As much as the guidelines are focused on genomic research, because of the interdependent nature of genetic research and genetic counselling, they may be useful for clinicians who conduct genetic counselling, too. Recommendations include ensuring that appropriate informed consent is sought and that there is capacity and expertise to support a proper feedback process during genetic counselling.

Genetic research plays an important role in producing results that support the scientific evidence on which sound genetics counselling is based. An approach proposed by Moher, Bouter, and Kleinert to the ethics of research is that, for society’s benefit, research and knowledge must be trustworthy (8). This implies that the assessment of the soundness of research and researchers’ ethics should include trustworthiness, rigor, and transparency. This has led to the development of the Hong Kong Principles (HKPs), which formed part of the 6th World Conference on Research Integrity proceedings (8). By extension, due to the interdependence of genetic research and genetic counselling, similar considerations could be applied to the process of genetic counselling. The assessment of the ethical aspects of genetic counselling could therefore also include the reliability and trustworthiness of the information being shared with patients and relatives. The processes of gathering informed consent that clinicians employ, as they engage patients during genetic counselling, should also be considered. Using clinicians with adequate levels of expertise to support patients during genetic counselling, becomes important. Furthermore, clinicians would need to be able to simplify information on the genetics of schizophrenia and similar illnesses. The use of diagnostic and classification systems like The Diagnostic and Statistical Manual of Mental Disorders and the International Classification of Diseases Manual, provides a universal concept of what schizophrenia is across different countries, cultures and contexts. This universal approach supports genetic counselling for schizophrenia in that the information on which the counselling is based is based on evidence from research on genetic causes of a similar entity across the difference practice settings at a global level (9, 10). Clinicians skilled in genetic counselling are critical in helping patients with the interpretation of evidence drawn from such genetic studies, a potentially complex exercise. An example is that the sharing of information on genetic variant (s) needs to be supported by strong evidence that demonstrates an association with and the causation of the condition in question, with reasonable accuracy and reliability of the clinical outcome (7).

Access to trained genetic counsellors who have the expertise to interpret complex genetic information and to provide feedback in a manner that empowers families to take decisions about their health outcomes and the management of their conditions, becomes critical (3, 4, 7). To ensure that the needs of communities where genetic counselling may be required are met, therefore, involves making provisions for those communities to gain assistance from trained and well-positioned experts. The expertise has to include not just scientific knowledge expertise on the subject matter but also the competence to negotiate complex ethical considerations as well as cultural competencies, among others, it is proposed.

In this paper, we examine several studies on the ethics of genetic counselling and the learnings from three large-scale multicentre studies on mental health and on the genetics of schizophrenia, to explore the ethical challenges that may confront clinicians. Linking the parallels between communication when conducting research on genetic causes of schizophrenia and communication when conducting genetic counselling for schizophrenia is done to illustrate similar challenges and how these might be addressed. This paper focuses on how to enhance the robustness of the ethical framework that governs such communication in both genetic counselling and genetic causes of schizophrenia research. This interrelatedness is important for building the evidence that forms the basis of knowledge to support genetic counselling.

## General concepts on ethical principles in genetic counselling

### Beneficence and non-maleficence

Some of the challenges in genetic counselling include balancing of the well-being and interests of the patient and family (beneficence) while avoiding causing harm (non-maleficence) through ensuring information sharing on genetic and hereditary risks is shared in a non-stigmatizing and empathetic manner. This balance may be further complicated by the lack of standardized guidelines; ambiguities related to variant interpretation due to a lack of translational studies; and underrepresentation of people of some communities in the reference genome (11). Although identifying rare variant risk factors for schizophrenia could have implications for the management and prognosis, current evidence has not reached the point of clinical application as yet (12). The matter is further complicated by the high heterogeneity of the African genome, when the context of Africa is taken into account (2–4, 12). In practical terms, uncertainty can affect the clinician’s ability to carefully balance beneficence and non-maleficence during genetic counselling, adding more to the complexities of clinical decision-making. A problem that clinicians face is that evidence about rare genetic variants that may play a role in schizophrenia is still preliminary and causality has yet to be established conclusively. The problem raises a question on whether it would be beneficial for patients to be given information when evidence is yet unable to provide more specific information about the genetic causes of schizophrenia, beyond the indication of a predisposition. There is also the possible risk of stigmatization that could be associated with the identification of such a genetic predisposition, raising the possibility that sharing this kind of information may also cause some degree of harm (13).

Clinicians thus need to weigh how much of the known factual information about heredity is shared for the benefit of patients while ensuring non-maleficence by supporting patients and families to manage the angst that comes with the uncertainty of not knowing the risk for developing schizophrenia in the offspring. Clinicians also carry the responsibility of supporting patients and families to balance the facts shared about the genetic risks while mitigating against the stigma that may be associated with the implication of passing on a chronic mental illness like schizophrenia to their offspring. A possible mitigating strategy that is proposed by Matimba et al. (7) is for clinicians to ascertain the validity of the results, their potential value and utility, as well as participant volition in order to determine the clinical validity of available evidence. This would then help to guide clinicians whether information on variants is worth sharing with patients, or not (7).

### Autonomy and informed consent

Challenges that may be faced by clinicians in an African setting may include difficulties in how to present complex ideas in a more simplified manner that is understood by patients and relatives during genetic counselling (7, 13). For consent to be considered truly informed, the participant needs to have a good understanding of the proposed interaction, intervention, or research, including how any related information will be stored and accessed and how it will be shared (14, 15). Moreover, the principle of autonomy requires that clinicians respect patients’ right to choose and make informed decisions without coercion. This adds another nuance to the process of informed consent during the engagement on genetic factors for schizophrenia (15). The implication for clinicians is that there is a need to weigh the patient’s capacity to consent and respect of their autonomy against the appropriateness and relevance of sharing information on the genetic causes of schizophrenia during genetic counselling. Factors like the patient’s mental stability at the time of disclosure of the clinical information relevant to genetic counselling are also important. The question of capacity to consent also introduces additional nuances on specific consent compared to broad consent and proxy consent, in cases where the patient does not have the capacity to consent (7, 15, 16).

Other challenges when it comes to informed consent for sharing and processing information about genetic causes of illnesses include the problem of simplifying the language of communication to make the jargon of genetic concepts like genes, DNA, and the different types of analysis or sequencing accessible to patients in general (14). Factors like the level of education, the understanding of basic biological concepts of genetics, scientific language development in contexts where the evolution of languages did not include common scientific terms, may all be additional obstacles (14, 15). This may limit the clinician’s ability to provide in-depth information on the genetics of schizophrenia, which can impact on the process of obtaining informed consent for divulging genetic predisposition for schizophrenia in the patient or relatives. The limitation may further impact on the process of obtaining informed consent and on what kind of information can be shared seamlessly with patients. Consent to divulge sensitive but important factors like the genetic predisposition for schizophrenia in the patient or in relatives may thus also not be easy to obtain.

In a country like South Africa, where there are 11 official languages and where only two of the 11 languages have a well-developed scientific language that includes commonly used terms to describe genetic concepts, these challenges are further amplified (7, 14, 15). Add to this the high levels of illiteracy in the largely unstudied communities in South Africa, and then one may potentially face ongoing obstacles to achieving the ideals of autonomy and informed consent during genetic counselling. The question clinicians and researchers are often faced with is whether informed consent is truly informed if patients only have vague ideas of the concepts being explored whilst their clinical information and data is being collected and accessed for research and the genetic counselling purposes.

### Confidentiality

Striking a reasonable balance between the contrasting approaches of sharing the minutest details about a study or genetic risk and glossing over such details to provide only the simplest information that participants or patients can understand during genetic studies or the process of genetic counselling may be difficult if the process is not guided by firm ethical boundaries. This is more so in the case of schizophrenia and other similar or severe mental illnesses, where capacity to give informed consent may not always be intact and confidentiality has to be balanced with the need to consult with the patient’s families or relatives. An example of how this was handled is presented by Campbell et al. (14), who were able to demonstrate that informed consent could be achieved in the genetic research of schizophrenia by the majority of participating patients that were approached to consider taking part in their study. The process of maintaining confidentiality was further supported through obtaining informed consent by using a formal assessment of capacity to consent. The formal assessments helped to maintain confidentiality in that they enabled more participants to provide informed consent directly, meaning that less of them ended up requiring proxy consent. A similar approach could be useful when communicating complicated technical concepts during genetic counselling in a clinical setting, thus ensuring there has been a good grasp of difficult concepts and creating a common understanding between the clinician, patient or relatives regarding what information will be shared during the process (14, 15).

### Distributive and social justice

Multisite, cross-country research, as a basis for evidence to support genetic counselling for schizophrenia, may also have implications with regard to access to resources for collaborating partners per country. The capacity to use multisite derived data in order to write and publish scientific papers drawn from these sites may not be feasible for all participating research units, depending on their resources (7). This would by extension affect availability of evidence on which to base the information shared during the genetic counselling process. Distributive justice and social justice become relevant in this context because of the effect that such resource limitations may have on availability of scientific evidence that would feed into genetic counselling, Data for this kind of research needs to be inclusive and clinicians an researchers need to ensure appropriate interpretation and cultural competency by local staff and teams. The inclusion of local authors could thus enhance regional relevance. Lack of equitable access to genetic research opportunities that support capacity building across genetic research and genetic counselling does not enhance the equitable development of expertise in the field of genetic counselling and may raise further obstacles to cultural competency in this field (3, 4). Local and regional expertise become important when working with or weighing data that may subsequently be used when conducting genetic counselling (3). McGuire et al. (16), argue that one of the core ethical challenges in modern genomics is rectifying inequities and attending to the imbalances in the access to resources to eliminate the privilege faced by some communities while others remain disadvantaged. The idea is that genomics in the future must be accessible to all, regardless of background or resources (16).

## The African context of ethical principles in genetic counselling

### Cultural competency and language

Cultural competency in mental health means the ability to engage with communities from different cultural backgrounds efficiently and in an appropriate and relevant manner. A clinician’s understanding of the socio-cultural factors balanced with the relevant medical issues is important to empowering patients to make sense of information shared on genetic aspects of the causes of schizophrenia (7, 17).

As in the consideration of distributive and social justice described above, regional nuances and cultural competence frameworks need to be taken into consideration when conducting research and interpreting data in multisite genetics and genetic counselling studies (3, 4, 11). Local authors, here too, become relevant as they may be better embedded within studied communities, as may be seen in a few examples of genetic research conducted in African communities (1–4).

When it comes to the language, especially in multilingual settings, the sharing of genetic counselling using relevant and accessible communication methods becomes important. Some studies have shown that basic terminology that may be used even during psychoeducation may not be understood by patients and relatives in multilingual setting such as South Africa (18, 19). Motlana et al. (18) found that, even in a context outside genetic research on schizophrenia and genetic counselling, terminology in local languages was important in the participants’ understanding of scientific concepts. Moreover, in a study on the communication of information on schizophrenia as a diagnosis to patients and their caregivers, Pooe et al. (19) describe that communication plans and materials drawn together with the patients and their families as end-users, are important, in producing relevant psychoeducational material. When communication of information on schizophrenia to patients and their families extends to the genetics and genetic counselling for the diagnosis, the difficulties may mount, due to the complexities of the information to be shared. This means that language difficulties, when coupled with cultural competence disparities, may further complicate genetic counselling on schizophrenia.

### Lessons from local and cross-country multisite studies

There have been a number of multicentre studies on the genetics of schizophrenia and the genetics of psychotic disorders (SAX, NeuroGap, Africa Consortium) which have included study sites in South Africa (1–3). There have also been other studies that have examined the process of setting up a mental health data bank for the purposes of research (20).

Each of these research projects has provided an opportunity to examine the process that researchers followed in addressing information sharing on genetic studies, informed consent issues for genetic research (NeuroGap, SAX) and the setting up of governance frameworks for gaining access to large scale data across country boarders (1, 2).

### The SAX study

#### What was the study about?

The SAX study is a case–control study that was conducted among Xhosa-speaking participants in South Africa (1). The goal was to identify genes important for schizophrenia by studying cases and controls from the South African Xhosa population. Data was collected from just over 1,800 participants (909 cases with schizophrenia and 917 controls). Whole-exome sequencing was used to assess DNA from participants and controls.

#### Approach

Recruitment took place in both rural and urban settings. A Community Advisory Board (CAB) was set up to represent community interests. A Community Advisory Board (CAB) was convened, and consisted of, patients and relatives advocacy groups, members of hospital facilities boards and other members of the local Xhosa communities. The CAB met once per quarter to discuss various issues arising from the project, including stigma. The approving Human Research Ethics Committees were updated annually, or when appropriate, on the proceedings of the CAB. The CAB also reviewed informed consent forms, where appropriate, for the purposes of guiding an update process based on the CAB input. Additional CAB activities included psychoeducation sessions about mental illness and local services to address the needs of people living with mental illness and their relatives.

#### Challenges

The main challenges had to do with communicating study information to potential participants effectively due to the need to translate the main study concepts around genetic research and the specific information regarding the DNA analysis that was to be conducted on collected samples. Limitations included the fact that the scientific language and everyday language to express the concepts and study information did not exist at the time of conducting the study. An added nuance was that grammatically correct translation from English to Xhosa was not always the most accessible language that could be easily understood by the community from which the participants would be recruited. Linked to the accessibility of information, when translated into Xhosa, was the issue of low literacy levels in some of the communities targeted for recruitment. This required alternative ways of communicating information other than the written format and also different ways of expressing informed consent beyond a signature on a consent form.

#### Lessons learnt

Using professional translators did not meet the required needs of the language challenges. The initial study information leaflets had to be reworked to adapt the first translated documents into more accessible language formats. This complicated the forward and backward translation in that it required a few rounds of translations to get to the desired quality and accuracy of information between the English and Xhosa participant information leaflet.

The issue of low levels of literacy was addressed through the verbal delivery of the study information and this was undertaken by research assistants and fieldworkers during outpatient clinic visits. The verbal deliveries allowed for a seamless introduction of the study and the opportunity to address questions in group settings. The group work was followed by a sharing of written information and an initial invitation that those who were interested in taking part in the study could indicate their interest to the fieldworkers. The next stage was to offer an appointment for enrolment where more detailed information about the study was shared, to those who were interested. Another important finding was that for a subsection of the study that sought consent for storage of cell line samples the recruiter who sought consent was a strong predictor of participant consent (15). This was even though there was standardization of the facts that were shared with participants. This implies that the interaction during sharing of information on genomics may be influenced by factors beyond just the scientific facts on genomics (15).

## The NeuroGap study

### Approach

This is a large scale case-control, multicentre study, across 4 countries in Africa namely, Ethiopia, Kenya, South Africa, and Uganda, that was still running at the time of the writing of the current paper. Participants were drawn from psychiatric inpatient and outpatient facilities. Genome-wide association studies were performed on DNA extracted from saliva. Data sharing occurred across country boundaries governed by country-specific pieces of legislation and regulations.

### Challenges

Informed consent procedures were affected by language limitations. Mitigation strategies included translation of the participant information leaflets, application of a formal assessment of capacity to consent tool and the use of outpatient waiting room engagements in group format to first lay the ground work for sharing information to potential participants.

### Lessons learnt

Adding an iterative element during the participant recruitment phase of the study enabled participants’ understanding of the study information to improve enough to be able to give informed consent for themselves instead of requiring proxy consent. Feedback on study results and findings on the genetic causes of schizophrenia was given to the community advisory board, the institutional managers and other stakeholders linked to the psychiatric services, also.

## The MindKind study

### Approach

The MindKind study researched how to include the participant voice by involving them in decision-making about the governance framework of the research project and about who should access the data bank for research purposes (20). The participants were further involved in how to set up a governance structure that is participant-led and in determining access to such data for researchers (MindKind).

### Challenges

Setting up advisory boards across the different countries differed according to access to resources, specifically smartphones and data for connectivity. This required specific remuneration for costs of data toward participants to be included within the proposal for the study and the budget. Study costs. The translation of jargon-filled technical terms having to do with the sharing and storage of data also posed a challenge.

### Lessons learnt

Critical to the inclusion of participants in the decision-making process adopted in the MindKind study was the upskilling of participants to empower them with the knowledge that enabled them to take an active role in such governance processes (18). Participants also understood the general terms and phrases that were used to explain technical concepts, even though there were no single words available as direct translations from English to the indigenous language of use.

## Discussion

The link between genetic counselling and genetic research in schizophrenia, as highlighted in the three studies described in this paper, seems to suggest that the ethical challenges clinicians face, in both contexts, share similarities. Concepts of beneficence and non-maleficence, distributive justice, informed consent, balancing accessible information against the potential for stigmatization, broad vs. specific content, and proxy consent, all require a balancing act that clinicians have to undertake in consultation with patients and relatives to ensure volition. When one adds the need for trustworthiness and reliability of the data shared with patients on the genetics of schizophrenia, then the clinical utility of the data being shared also comes into play. Persistent challenges include the lack of equity in the availability of and the access to resources, as well as the limited access to clinicians with expertise in genetic research and genetic counselling. Although these persistent challenges may imply that clinicians may continue to struggle to meet community needs on genetic counselling for schizophrenia, there are some encouraging signs of potential for growth and capacity building. Evidence of opportunities is further outlined next, using a global and regional perspective as well as evidence from genetic counselling conducted across other medical specialities.

### The global context

The possibility of stigmatization when informing individuals and families about their genetic risk for schizophrenia has already been alluded to and remains a significant factor to consider (13). Further, there may be special population issues, e.g., the testing of minors when assessing for the risk of schizophrenia. The decision to disclose the potential for undesirable and poor future outcomes may add to stigma and other negative consequences and psychosocial effects on the child (21). Another nuance to the potential for stigmatization could be ethical concerns about potential for discrimination through exclusion from health insurance (15). These concerns are highlighted because of the potential for stigmatization of individuals who are identified as having an increased genetic risk for schizophrenia, as well as the psychological impact of knowing that one may develop schizophrenia later in life (13). Issues of confidentiality and privacy may arise as genetic information could be used, misused or disclosed in desirable and unforeseen ways (13). Given the potential psychosocial impacts of these challenges, one approach clinicians could take is to balance the consequences of sharing the information with the patient or relatives with the potential effect this could have on the individual and the family should they not be afforded the opportunity to manage the genetic risk. Examples of managing such genetic risks could include taking pre-emptive steps to manage stress effectively, increase psychosocial support and avoid substance abuse, as these factors could compound the genetic vulnerability. These remain universal approaches irrespective of the region of the world.

Use of the Genetic Counselling Outcome Scale (GCOS-24), which is a Patient Reported Outcome Measure (PROM), has been advocated to help provide evidence to support service development in genetic counselling. This measure was developed for use in the National Health Service (NHS) system of healthcare in The United Kingdom and has since been validated across other countries like Denmark and The Netherlands (22–24). The other factor to consider is what patients and parents may find useful to know in the clinical context. A cross country study in Canada and Britain was able to establish that parents valued information on causal diagnoses and information on management focused on improving health and wellbeing. There was also a wish for the focus of studies to be on finding further evidence on which to base information shared to decrease uncertainty (25).

Pollard et al. (26) also noted barriers to the implementation of psychiatric genetic counselling services in general which include the complexities of uncertainty in psychiatric diagnoses, patient engagement and ethical concerns regarding limited capacity.

Jenkins and Arribas-Ayllon (27) described challenges in genetic counselling encountered in an Arab subpopulation of participants which included 2020, a lack of knowledge about genetic counselling in the target population. This contributed to leading to negative preconceptions of genetic testing, feelings of uncertainty, frustration, and distrust. Religious subgroups had different attitudes toward genetic counselling. There was also miscommunications between healthcare practitioners and patients from minority ethnocultural groups, further highlighting the importance of cultural competence training in genetic counsellors to enhance autonomy in decision making (27).

### The African and South African context

In African societies, genetic counselling for schizophrenia may be faced with additional unique ethical challenges. These may include issues of access due to resource limitations, informed consent, and the impact of collective values and practices on individual autonomy (28). The collectivist nature of some African societies, where the individual is expected to prioritize the welfare of the group over individual autonomy may affect the concept of informed consent. This may mean that families may make decisions collectively, often citing the concept of ubuntu and a need to discuss important health concerns with an elder in the family (21, 28). There is also the issue of the availability and affordability of genetic testing in African settings, which may limit access to genetic counselling services (21, 28).

In South Africa, genetic counselling for schizophrenia is similarly also faced with additional unique ethical challenges (7, 29). The country has a complex history of racial, economic and social inequality, which may further impact access to genetic counselling services (21). Learnings from the MindKind and SAX studies that support the inclusion of a representative community voice to guide the governance framework of genetic research may similarly be applied to genetic counselling through the formation of community advisory boards (CABs). Some of the challenges already highlighted could be managed by setting up these boards to be consulted by, and to collaborate with clinicians, in developing practice guidelines on genetic counselling for mental disorders (1–3, 20, 21). In that way cultural practices and social norms could be taken into account, with region-specific emphasis allowed to shape and influence the guidelines.

Another area for future development in populations with diversity in culture and languages, as found in South Africa, is the issue of translating key genetic concepts into indigenous languages. The lessons learnt from the studies like the NeuroGap and SAX, present opportunities to amend practice and training and make it work for the scientific community by ensuring that gaps are addressed and plugged. To maximize on lessons learnt from the studies illustrated above, clinicians and researchers can use these gaps as opportunities to amend practice or training and improve access to genetic counselling by ensuring that such gaps are addressed and plugged through training. Learnings from the NeuroGap Study indicate that one way around the difficulty of translating information on genetics may be to deliberately conceptualize and write participant info leaflets in indigenous languages first and then translate these to English, which may at times be an easier process to undertake than the other way round (2).

### Genetic counselling in other specialities

Useful examples of genetic counselling may be found in other specialities and conditions like pulmonary arterial hypertension as described by Morris et al. (30). They investigated the development of a genetic counselling service in a national referral center to meet the needs of patients and relatives. This included setting up a screening and monitoring service to meet the affected individuals and families during the asymptomatic stages instead of waiting until the development of symptoms in those identified to carry the genetic vulnerability. A study on the hereditary aspects of renal carcinoma and genetic counselling in Canada also highlighted the need to provide a timely diagnosis and effective treatment and how this was dependent on the availability of appropriate genetic testing and genetic counselling for patients. When genetic testing was linked to screening, efficacy was demonstrated in the clinical utility and application of this information to help patients and relatives manage their genetic risk (31).

## Recommendations and opportunities for improving genetic counselling

Recommendations and opportunities are presented at systemic and clinical practice level. Training of genetic counsellors is required to improve capacity and expertise in the field of genetic counselling in schizophrenia and other psychotic syndromes. Given the many challenges that have already been outlined as risks to access to care and culturally appropriate and locally relevant information, guidelines specific to genetic counselling also need to be developed to fit regional and national community need (3, 5, 7, 15). Community advisory boards need to be included as a norm in genetic research, so as to ensure that the data interpretation takes into account relevant social cultural aspects which may otherwise not be included (1–3). Investing resources in capacity building initiatives that ensure availability of culturally competent genetic counsellors represents an opportunity that can potentially make a difference to progress in the creation and discovery of scientific knowledge on the genetic causes of schizophrenia. A well-informed community from which participants are drawn is likely to be empowered through the enhancement of access to reliable and trustworthy information, in a context that respects multilingual expressions.

Training in genetic research and genetic counselling needs to be expanded into undergraduate medical and other health professions training programs, to further expand expertise, especially in under-resourced communities. The diversification of training is important especially in under-resourced communities, as the liberty to have standalone genetic counsellors is not a luxury all can afford (32, 33) The format of training in South Africa for those who do genetic counselling in psychiatry and schizophrenia, more specifically would also need to be made fit-for-purpose in specific regions. The qualification in South Africa and other African countries has great potential and opportunities for development because it is a relatively young field of specialization—especially in the context of accreditation of fit-for-purpose courses and qualifications. Good quality outcomes as well as sound ethical practice and evidence-based scientific approaches can be enhanced in the context of training culturally-competent counsellors.

Kim et al. (34) further advise that non-clinical geneticists need more clinical knowledge and experience to counsel patients regarding genetic testing results, to help address complexities that may contribute to adverse outcomes for patients and their families. Nagy et al. (35) also advocates that recent findings in genetic research must lead to profound changes in genetic and family counselling in schizophrenia. It is equally essential for clinicians to ensure a balanced approach that weighs the interplay between genetic and other risk factors which include social determinants and environmental risk factors (35). This can empower patients with schizophrenia and their families to make well informed decision during the process of genetic testing and counselling and could also help to manage unrealistic expectations, discrimination and stigma related to incomplete understanding of genetic test results (36).

There are limited clinical practice guidelines to help clinicians to weigh the role of genetic testing in mental disorders like Schizophrenia. What complicates this is the complex interaction between genetic and environmental factors (37). This limitation in clinical practice guidelines may be addressed through the use of the colored beads in a jar model, which was developed and trialed in Canada (37). The colored beads in a jar model helps to conceptualize and convey the complex interplay between genetics, social determinants and environment for clinicians and patients (37). In the model, genetic and environmental contributors to manifestation of the condition being discussed are represented by different colored beads in a jar. The concept of genetic loading and its contribution to vulnerability to the condition is further explained using different permutations and differing amounts of the colored beads to demonstrate what degree of the disorder is thought to be due to genetic factors and what degree of the disorder is due to other factors, including environmental factors (37).

Norms need to be developed to address the translation of basic concepts on genetic information into indigenous languages. The maintenance of good quality translation for diverse populations and end-users could be extended beyond a condition such as schizophrenia and psychiatry but could encompass other disciplines within which training and accreditation bodies like the Health Professions Council of South Africa (HPCSA) and similar bodies across the global South and the North, could expand (32). Such norms can be a basis for guidelines for the setting of minimal information and concepts that need to be covered in patient information and participant information leaflets in the context of genetic counselling or genetic research. Inclusivity of genomic studies would address the exclusion of under-resourced communities and ensure a more distributive, equitable and socially just representation of populations in the ongoing mapping of genetic vulnerability to illnesses (38). To expand on these opportunities, commitment is required from different key stakeholders like the scientific community, academic institutions, government funding initiatives and medical insurance companies, to fund resources and support capacity-building initiatives. Table 1 provides a summary of challenges an recommendations.

**Table 1:** A summary of some of the challenges and recommendations.

Challenges	Recommendations
Ethical challenges linked to nuances of culture and language differences	Establish Community Advisory Boards and collaborate to develop norms and guidelines for supporting communication with local communities
Ethical challenges to do with confidentiality vs. sharing of genetic risk	Set up a genetic assessment, screening and monitoring service to support families who have been identified to carry the genetic vulnerability
Lack of capacity and resources to meet patient, relatives or participant needs	Develop norms and guidelines for clinical practice
	Be more inclusive in participant sampling to target under-resourced and underrepresented communities to ensure mapping of genetic vulnerability is inclusive
	Get to know the language and cultural intricacies that may be included to tailor the information packaging and sharing of information on genetics of schizophrenia Work with local community representatives and partner to find ways to make the technical language used in genetic counselling more accessible
	Systemic Formation of key collaborative partnerships between the scientific community, academic institutions, government funding initiatives and medical insurance companies to ensure adequate funding for increasing research and clinical expertise in genetic counselling
	Training of genetic counsellors
	Borrow from the research on genetic counselling to also form community advisory boards who can support the development of local specific language and capacity to support the sharing of information process during genetic counselling
	Include different forms of communicating info on genetic counselling to include written, verbal, visual and other accessible means of sharing relevant information with patients and relatives as well as with participants in genetic research
	Telemedicine principles could be applied in genetic counselling to enhance capacity and access culturally-competent genetic counsellors for far flung areas where resources may be limited

## Conclusion

At a glance, prevailing challenges that face clinicians in genetic counselling for schizophrenia, and the persistent resource limitations may seem discouraging. However, opportunities exist for progress to be made in building ethically sound technical capacity and expertise in genetic counselling for schizophrenia and other mental disorders. Commitment from key stakeholders will be essential to ensure availability of funding and resources to advance and support development of expertise in genetic counselling. Collaborative opportunities to improve access to resources could translate to increased capacity that may address challenges and help develop more expertise in this field. Funding and creation of training opportunities could further strengthen accessibility when such opportunities are leveraged to target under resourced communities. If one maximizes on all these opportunities successfully, the field of genetic counselling in schizophrenia and other mental disorders has significant potential and possibilities for expansion to ensure the goal of accessibility for all who need this service is realized.

## Data availability statement

The original contributions presented in the study are included in the article/supplementary material, further inquiries can be directed to the corresponding author.

## Author contributions

All authors listed have made a substantial, direct, and intellectual contribution to the work and approved it for publication.

## Conflict of interest

The authors declare that the research was conducted in the absence of any commercial or financial relationships that could be construed as a potential conflict of interest.

## Publisher’s note

All claims expressed in this article are solely those of the authors and do not necessarily represent those of their affiliated organizations, or those of the publisher, the editors and the reviewers. Any product that may be evaluated in this article, or claim that may be made by its manufacturer, is not guaranteed or endorsed by the publisher.
